# *Staphylococcus aureus* Modulates Carotenoid and Phospholipid Content in Response to Oxygen-Restricted Growth Conditions, Triggering Changes in Membrane Biophysical Properties

**DOI:** 10.3390/ijms241914906

**Published:** 2023-10-05

**Authors:** Laura Zamudio-Chávez, Elizabeth Suesca, Gerson-Dirceu López, Chiara Carazzone, Marcela Manrique-Moreno, Chad Leidy

**Affiliations:** 1 Biophysics Group, Physics Department, Universidad de los Andes, Bogotá 111211, Colombia; ld.zamudio@uniandes.edu.co (L.Z.-C.); e.suesca87@uniandes.edu.co (E.S.); 2 PhysCheMath Research Group, Chemistry Department, Universidad de América, Bogotá 111211, Colombia; gerson.lopez@profesores.uamerica.edu.co; 3 Laboratory of Advanced Analytical Techniques in Natural Products (LATNAP), Chemistry Department, Universidad de los Andes, Bogotá 111211, Colombia; c.carazzone@uniandes.edu.co; 4 Chemistry Institute, Faculty of Exact and Natural Sciences, University of Antioquia, Medellin 050010, Colombia; marcela.manrique@udea.edu.co

**Keywords:** *Staphylococcus aureus*, biofilms, staphyloxanthin, LC-DAD-APCI-MS/MS, phospholipids, LC-ESI-MS/MS, fluorescence spectroscopy, infrared spectroscopy

## Abstract

*Staphylococcus aureus* membranes contain carotenoids formed during the biosynthesis of staphyloxanthin. These carotenoids are considered virulence factors due to their activity as scavengers of reactive oxygen species and as inhibitors of antimicrobial peptides. Here, we show that the growth of *S. aureus* under oxygen-restricting conditions downregulates carotenoid biosynthesis and modifies phospholipid content in biofilms and planktonic cells analyzed using LC-MS. At oxygen-restrictive levels, the staphyloxanthin precursor 4,4-diapophytofluene accumulates, indicating that the dehydrogenation reaction catalyzed by 4,4′-diapophytoene desaturases (CrtN) is inhibited. An increase in lysyl-phosphatidylglycerol is observed under oxygen-restrictive conditions in planktonic cells, and high levels of cardiolipin are detected in biofilms compared to planktonic cells. Under oxygen-restriction conditions, the biophysical parameters of *S. aureus* membranes show an increase in lipid headgroup spacing, as measured with Laurdan GP, and decreased bilayer core order, as measured with DPH anisotropy. An increase in the liquid–crystalline to gel phase melting temperature, as measured with FTIR, is also observed. *S. aureus* membranes are therefore less condensed under oxygen-restriction conditions at 37 °C. However, the lack of carotenoids leads to a highly ordered gel phase at low temperatures, around 15 °C. Carotenoids are therefore likely to be low in *S. aureus* found in tissues with low oxygen levels, such as abscesses, leading to altered membrane biophysical properties.

## 1. Introduction

*Staphylococcus aureus* (*S. aureus*) is an opportunistic pathogen; a Gram-positive, facultative anaerobic bacterium, naturally present in human skin and mucous membranes [1,2], which can also invade and reside in the interior of host cells, including human keratinocytes [3]. *S. aureus* is responsible for multiple hospital-acquired diseases such as pneumonia, different kinds of human skin infections, osteomyelitis, endocarditis and sepsis, presenting an important health risk worldwide [1]. Due to the emergence of multiple-drug resistant strains [4], understanding the physiology of this bacterium is crucial in the development of complementary treatment strategies. 

*S. aureus* has evolved the ability to modify the lipid composition of its membranes in response to environmental stresses such as oxidative stress, osmotic stress, and the presence of antimicrobial agents [5,6,7,8], as well as during changes in *S. aureus* growth conditions [6,8]. Modifications in phospholipid headgroup and acyl chain composition or carotenoid content influence the biophysical properties of *S. aureus* membranes [6,9], leading to changes in membrane rigidity and lipid packing [6,8,10,11]. 

The biosynthesis of carotenoids in *S. aureus* has been identified as an important virulence factor leading to resistance to antimicrobial peptide activity and to reactive oxygen species (ROS) generated during the immune response. Carotenoids also act as scavengers of free radicals (hydroxyl OH) generated during oxidative stress or during cellular respiration [12]. The production of different carotenoid species in *S. aureus* is mainly associated with the biosynthesis of staphyloxanthin (STX). The STX biosynthetic pathway begins with the dimerization of two farnesyl diphosphates to form 4,4-diapophytofluene (4,4′-DPE) [8]. This is followed by a series of dehydrogenase reactions catalyzed by 4,4′-diapophytoene desaturases (CrtN). Oxidation reactions then lead to the formation of 4,4′-diaponeurosporenoic acid (4,4′-DNPA). The carboxylic acid is then functionalized with a glucose, through CrtQ, and a fatty acid with a varying acyl chain is finally attached, through CrtO, to form STX [8,13]. Variations in oxygen levels have been shown to modulate this biosynthetic path [14]. Due to a series of conjugated double bonds, the rigid structure of the diaponeurosporenoic acid leads to increased membrane rigidity, which confers on *S. aureus* a higher resistance to antimicrobial peptides [15]. This is in addition to its protective role as a free radical scavenger [8,13,16,17,18]

The main phospholipid components in *S. aureus* membranes are the anionic phospholipids phosphatidyl-glycerol (PG) and cardiolipin (CL), as well as the cationic phospholipid lysyl-phosphatidylglycerol (Lys-PG), which are present in the membrane with varying acyl chain lengths [6,10,19]. Phospholipid acyl chains are mainly saturated or branched in *iso-* and *anteiso-* conformations, where the branched/saturated ratio helps modulate the level of lipid packing in the membrane [6,20]. CL is an anionic tetra-acyl chained glycerophospholipid, which has been shown to increase lipid packing levels [21], modulate membrane curvature [22], and confer lipid bilayer membranes with increased mechanical resistance towards antimicrobial peptides [21,23]. The MprF-mediated biosynthesis of the cationic lipid Lysyl-PG modulates the surface charge of the membrane, decreasing the anionic surface charge, and leading to a reduction in the affinity of antimicrobial peptides, therefore inhibiting their lytic activity [9].

*S. aureus* can invade different tissues in the host, where oxygen levels vary widely [24], showing the capacity to live at low oxygen levels [25] or even under fully anaerobic conditions in certain tissues such as abscesses [26]. For this reason, *S. aureus*, as a facultative anaerobic bacterium, relies on switching its metabolism from aerobic respiration, in the presence of excess oxygen, to a combination of fermentation and anaerobic respiration, using alternative electron acceptors such as nitrate, at low oxygen levels [24]. This switch in metabolism at oxygen-restrictive levels leads to lower ATP production and slower growth. One of the main roles that carotenoids play is as scavengers of free radicals, which are produced in high quantities at high oxygen levels. A two-component AirSR oxygen response regulator in *S. aureus* has been reported to positively regulate the STX biosynthetic pathway operon *crtOPQMN* in the presence of increased oxygen levels, showing the importance of STX biosynthesis during oxidative stress [14]. The downregulation of STX production at low oxygen levels may help reduce metabolic costs under anaerobic respiration or fermentation, since the need to scavenge oxygen free-radical species will be reduced.

In the present study, *S. aureus* was grown either as biofilms or under planktonic conditions, exploring how the membrane lipid composition was modulated under restrictive and non-restrictive oxygen levels. Since carotenoid production in *S. aureus* is closely tied to available oxygen levels, we mainly focused on changes in carotenoid content as determined via liquid chromatography coupled to mass spectrometry (LC-MS), reporting on overall levels of carotenoids and the proportion of STX precursors for the different growth conditions. We also report on changes in phospholipid headgroup composition, showing shifts in the proportion of PG, CL, and Lysyl-PG phospholipids under restrictive oxygen growth conditions. These shifts in lipid composition alter the biophysical properties of *S. aureus* membranes. Changes in lipid headgroup spacing measured with Laurdan Generalized Polarization (Laurdan GP), bilayer core dynamics measured with DPH anisotropy, and overall lipid phase behavior measured with Fourier transform infrared (FTIR) spectroscopy are reported. Scanning electron microscopy images are used to evaluate changes in biofilm and planktonic average cell size and changes in the production of exopolysaccharides in biofilm cells. Oxygen levels vary greatly for different body parts and tissues [27]. For this reason, understanding how *S. aureus* lipid composition is affected by oxygen, and how bacterial membrane biophysical properties are affected by these shifts, may be relevant in understanding the susceptibility of *S. aureus* in different tissues to treatments involving oxidative stress or antimicrobial peptide treatments. 

## 2. Results

### 2.1. Cell Morphologies in Biofilm and Planktonic Cells and Production of Exopolysaccharides in Biofilms

**Figure 1:**
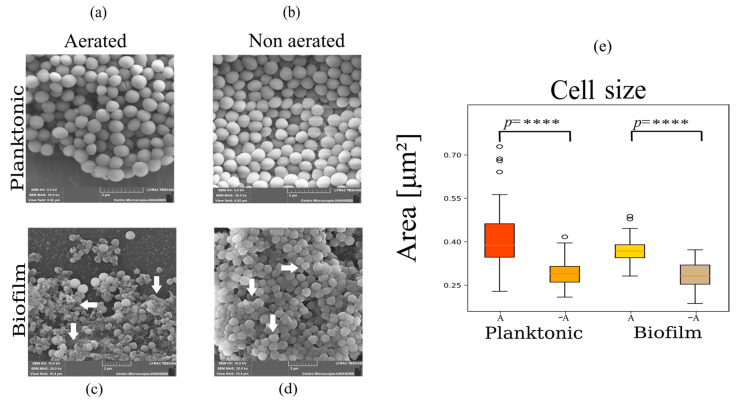
Scanning electron microscopy images show changes in cell morphologies for planktonic (**a**,**b**) and biofilm cells (**c**,**d**) grown under different aeration conditions. Aerated biofilms show variations in cell morphologies, the production of exopolysaccharides, and the presence of damaged cells, as pointed out by the arrows in (**c**). Non-aerated biofilms show tightly packed cells covered by a smooth exopolysaccharide structure as pointed out by the arrows in (**d**). (**e**) Boxplots showing the mean area of the cells, where open circles show the dispersal of the data points. These data show significant difference in the size between the different growth conditions (**** *p* < 0.0001).

Images obtained with scanning electron microcopy (SEM) are shown in Figure 1 for planktonic cells and biofilms under different aeration conditions. In Figure 1c,d, the formation of biofilm structures, characterized by an increase in exopolysaccharide structures, is evident in the images for both aerated and non-aerated conditions. However, exopolysaccharides are more prevalent for aerated biofilms (Figure 1c). Biofilms can be found in different stages of development [28,29], which are the initial attachment where the planktonic cells attach to surface due to electrostatic interaction, the growth phase where cells will begin to divide and accumulate to obtain a mature biofilm, and finally the detachment stage where cells are dispersed over the catheter. The biofilm images shown in Figure 1c,d are likely in different growth stages due to the amount of biomass that is observed in the sample, where the non-aerated samples showed more biomass. 

Analysis of cell size, measured as area per cell, showed a statistically significant reduction in cell size under non-aerated conditions for planktonic and biofilm growth states (Figure 1e and Table 1). This is likely induced by a switch from aerobic to anaerobic respiration and upregulation of fermentation which has a lower ATP yield [24]. 

Additionally, exopolysaccharide structures differed between aerated and non-aerated samples for biofilms. In the case of biofilms fed with aerated media, images show dispersed cells embedded in a rugged and extensive exopolysaccharide structure (Figure 1c), while under oxygen-restricting conditions, biofilms showed densely packed cells closely surrounded by a smooth exopolysaccharide structure (Figure 1d).

### 2.2. Biofilms Pigmentation Levels Vary When Exposed to Oxygen Stress

**Figure 2:**
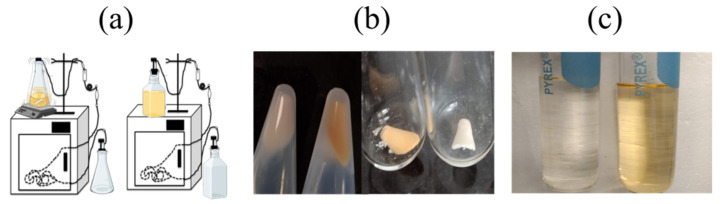
Biofilms lose pigmentation levels in response to oxygen restriction. (**a**) Experimental setup for biofilm formation in the presence (left) and absence (right) of aeration. Incubation temperature is maintained at 37 °C. (**b**) Biofilm obtained from the catheter before (left) and after (right) lyophilization, showing variations in the pigmentation levels. (**c**) Lipid sample after extraction; the lighter-colored sample is the biofilm grown under low aeration conditions (left). Biofilms grown in the presence of higher aeration (right) levels show intense pigmentation.

For planktonic cells, significant differences in pigmentation were detected by modifying agitation levels (Figure 2). Total lipid extractions were obtained from these samples. The oxygen-restricting culture always showed a lower OD than the aerated culture which agrees with a lower cell count, as expected under non-aerated conditions. In the case of biofilms, a variation in the experimental set up, introducing aeration of the feeding media (Figure 2a), induced changes in cell pigmentation and biomass, showing a remarkable loss in pigmentation and reduction of biofilm mass under oxygen-restricting conditions (Figure 2b,c). Total lipids were extracted from the samples following the protocol in [6,8]. Lipid yields for biofilm samples were significantly lower due to the high amount of protein in the samples.

### 2.3. Carotenoid and Phospholipid Content and Composition

**Figure 3:**
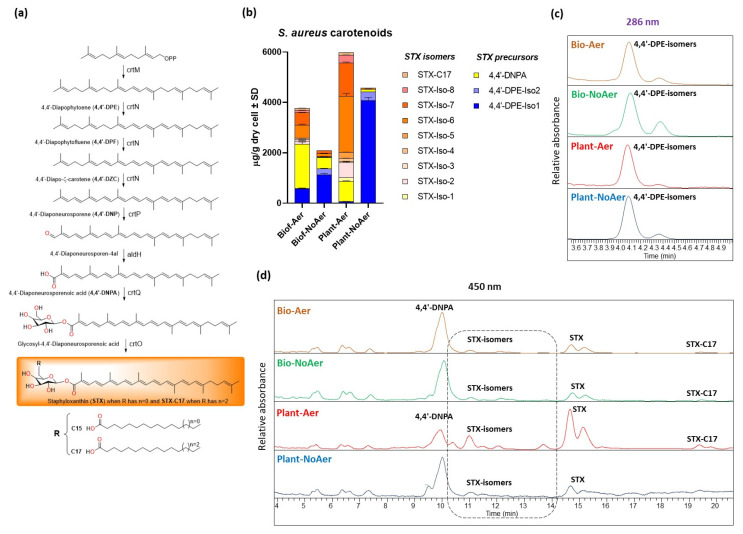
Carotenoids analyzed using LC-DAD-APCI-MS/MS from biofilms and planktonic *S. aureus* cells. (**a**) Biosynthetic pathway for carotenoids in *S. aureus* (**b**) Carotenoid concentration in μg/g dry *S. aureus* cell. (**c**) Chromatogram of 4,4′-Diapophytoene isomers at 286 nm. (**d**) Chromatogram of carotenoids at 450 nm.

Carotenoid content and composition was analyzed with liquid chromatography-diode array detection-atmospheric pressure chemical ionization-tandem mass spectrometry (LC-DAD-APCI-MS/MS) for all growth conditions, and the results are presented in Figure 3 and Table 2. The three chemical species that were evaluated were 4,4′-DPE, a precursor of STX that results from the dimerization of two farnesyl diphosphates; 4,4′-DNPA, which presents an extensive conjugated double bond structure, but is not functionalized with glucose; and the different STX species varying in the chain length of the fatty acid group attached to the glucose (Figure 3a) [8]. We observed a significant reduction in the overall amounts of STX species produced in the cells reported as µg/g dry cell weight (orange pallet in Figure 3b) under oxygen-restricting conditions. The precursor species 4,4′-DPE (Figure 3a,c) greatly accumulates in planktonic cells under oxygen-restricting conditions (blue pallet in Figure 3b), indicating that the dehydrogenation reaction catalyzed by 4,4′-diapophytoene desaturases (CrtN) is inhibited when oxygen is restricted. Low concentrations of 4,4′-DNPA and STX (Figure 3b,d) were also found for these conditions. Accumulation of 4,4′-DPE was also observed in non-aerated biofilms, but to a smaller degree. 4,4′-DNPA appears to accumulate more prevalently in biofilms at the expense of STX compared to planktonic cells (yellow in Figure 3b). This may be a result of reduced levels of available glucose in the biofilm growth phase. 

**Figure 4:**
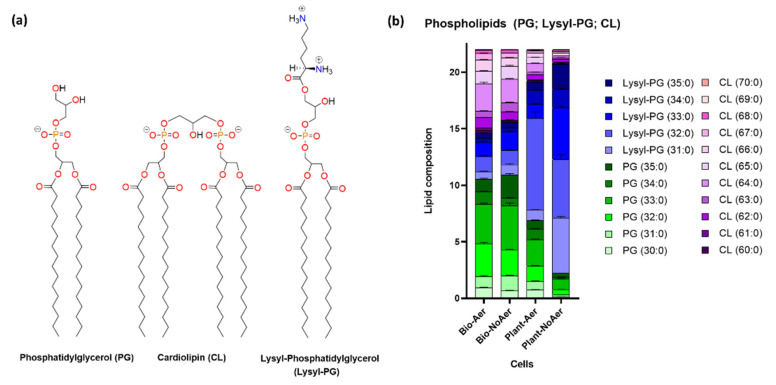
(**a**) Molecular structures of main phospholipid components. (**b**) Lipid analysis by LC-ESI-MS/MS shows shifts in the proportions of PG, CL, and Lysyl-PG for the different growth conditions. An increase in the proportion of Lys-PG is observed for planktonic *S. aureus* cells under oxygen-restricting growth conditions.

Figure 4 and Table S1 shows an analysis of the main phospholipid components of *S. aureus*, PG, CL, and Lysyl-PG for biofilms and planktonic cells. The chemical structure of the three phospholipid species is shown in Figure 4a. PG is an anionic phospholipid with the negative charge in the phosphate group at physiological pH with a glycerol attached at the headgroup side of the molecule. CL has two anionic phosphates attached through a glycerol group, and a total of four acyl chains. 

When comparing biofilms with planktonic cells (Figure 4b), the results show an increase in the proportion of CL in the membranes of biofilm cells compared to planktonic cells. Phospholipid composition in biofilms is observed to be independent of aeration levels (Figure 4b). In contrast, planktonic cells show an increase in the proportion of Lysyl-PG under oxygen-restricting growth conditions compared to aerated planktonic cells. The mass spectra of PG, Lysyl-PG, CL, and STX are presented in Figure S1.

### 2.4. Biophysical Properties of Membranes

The biophysical properties of the bilayers under different growth conditions were probed using a variety of techniques in order to obtain an integrated view of the effect of carotenoid production on membrane properties. This is performed as a function of temperature to assess the gel to liquid-crystalline phase transitions that are present in *S. aureus* membranes. At lower temperatures, the lipid bilayer is normally in the gel phase regime, which presents increased packing and rigidity [30]. At higher temperatures, the membrane enters the liquid-crystalline phase regime, which has higher levels of acyl chain disorder and decreased headgroup packing (among other parameters that change). The liquid-crystalline phase is considered to be the physiologically functional phase that allows the bacteria to undergo metabolism and cell division. 

Laurdan GP fluorescence spectroscopy was used to detect changes in lipid headgroup spacing for the different samples as a function of temperature. Laurdan is a fluorescent molecule with an emission spectrum that is sensitive to the polarity of the surrounding environment, resulting in a red-shifted emission spectrum when the polarity increases. This shift was measured by calculating the Laurdan GP parameter (see methods). The fluorescence moiety of Laurdan positions itself at the headgroup/tail region of the bilayer. A reduction in GP is associated with increased hydration of this region due to an increase in headgroup spacing. For this reason, Laurdan probes lipid headgroup spacing, which decreases when lipid packing increases.

**Figure 5:**
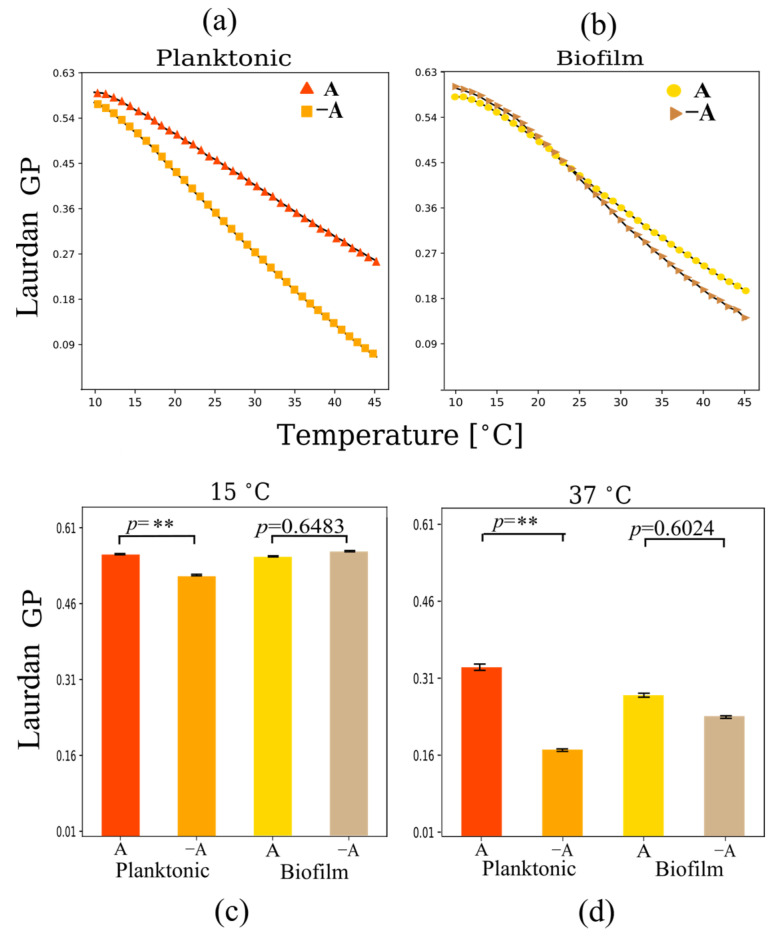
Laurdan GP polarization as a function of temperature for multilamellar vesicles from lipid extracts of (**a**) planktonic and (**b**) biofilm systems. Changes in Laurdan GP at (**c**) 15 °C and (**d**) 37 °C under planktonic and biofilm growth conditions in the presence and absence of aeration. Planktonic cells show a reduction in headgroup spacing under aerobic conditions. Measurements were performed in triplicate. Error bars in (**a**,**b**) are smaller than the symbols. ** *p* < 0.01.

All measurements showed an increase in headgroup spacing as temperature increases, evidenced by a reduction in Laurdan GP with temperature (Figure 5a,b). This is consistent with the membranes entering the liquid-crystalline phase, which is characterized by increased headgroup spacing. In Figure 4c,d, the Laurdan GP values are compared in the gel phase regime (15 °C) and the liquid-crystalline phase regime (37 °C). 

In relation to aeration conditions, both biofilm and planktonic cells showed higher levels of Laurdan GP under aerated conditions at physiological temperatures (37 °C), indicating an increase in lipid packing related to a reduction in headgroup spacing as carotenoid concentration increases (Figure 5c,d). However, biofilms do not show notable differences in GP. The data indicate that non-aerated biofilms show high levels of lipid packing even in the absence of carotenoids. Only planktonic cells show significant differences in headgroup spacing in the presence of carotenoids, and these differences are relatively small, indicating that carotenoid content does not affect significantly headgroup spacing. 

**Figure 6:**
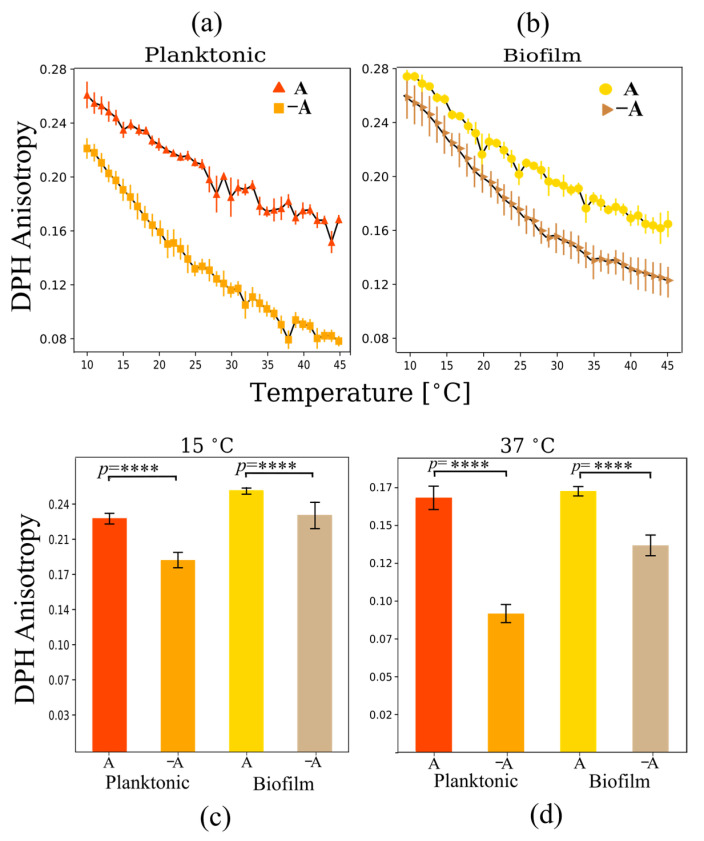
DPH anisotropy as a function of the temperature for multilamellar vesicles from lipid extracts of planktonic and biofilm systems for (**a**) planktonic and (**b**) biofilm growth states. Changes in DPH anisotropy at (**c**) 15 °C and (**d**) 37 °C under planktonic and biofilm growth conditions in the presence and absence of aeration. The anaerobic samples showed lower levels of lipid packing in the liquid-crystalline phase temperature regime. **** *p* < 0.0001.

DPH fluorescence anisotropy was used to explore the level of disorder at the bilayer core for the different growth conditions [31]. When DPH is excited with polarized light, the molecule also emits polarized light. However, DPH light polarization is lost after excitation if the tumbling rate of the molecule is high compared to the excitation lifetime. A reduction in DPH fluorescence polarization anisotropy indicated high DPH tumbling rates, and therefore high levels of disorder at the bilayer hydrophobic core, where DPH is positioned. DPH anisotropy results also showed a decrease with temperature for all samples, which is consistent with the increased level of acyl-chain disorder in the liquid-crystalline phase compared to the gel phase (Figure 6a,b). Again, a cooperative melting event is not evident from the DPH thermograms. 

In contrast with Laurdan GP, DPH anisotropy changes significantly in the presence of carotenoids induced by aeration for both planktonic and biofilm samples. The increase in order at the membrane core occurs both in the gel phase regime at 15 °C and in the liquid-crystalline regime at 37 °C (Figure 6c,d). This is consistent with previous results that show that the presence of carotenoids increases DPH anisotropy levels at 37 °C [6]. In general, the presence of carotenoids increases membrane rigidity at the bilayer core [6,8,32].

Fourier transform infrared spectroscopy (FTIR) is used to monitor the symmetric stretching vibration (vsCH_2_) of the acyl chains in the bacterial membrane [33]. The wavenumber presents a sharp increase when the membrane transitions from the gel phase to the liquid-crystalline phase, as the number of +gauche and -gauche rotomers increases in relation to the trans rotomers when the acyl chains become disordered. An increase in the number of gauche rotomers compared to the trans configuration results in an increase in the vsCH_2_. Therefore, this vibrational parameter is an indicator of the level of acyl-chain disorder in the bilayer membrane. FTIR is also very useful in detecting the cooperative melting event in membranes, as the vsCH_2_ vibration undergoes a cooperative shift at the melting temperature [34].

**Figure 7:**
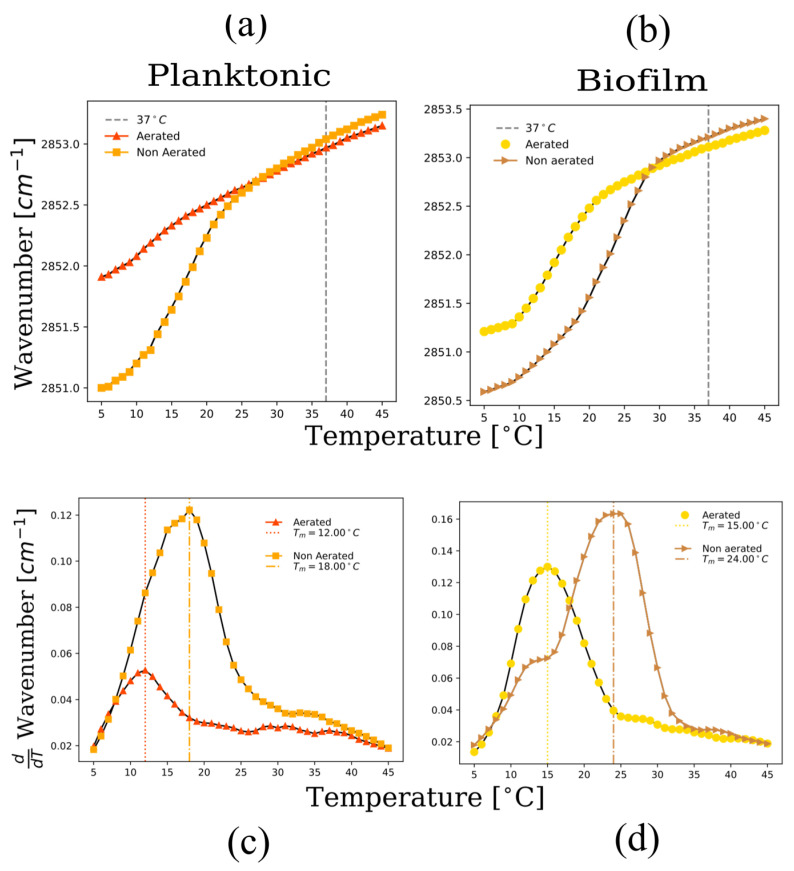
FTIR spectroscopy as a function of temperature for multilamellar vesicles from lipid extracts of planktonic and biofilm systems. Peak position of the symmetric CH_2_ stretching vibration band of the acyl chains as a function of temperature for (**a**) biofilms, and (**b**) planktonic cells under different aeration levels. In (**c**,**d**) the first derivative of the wavenumber as a function of temperature is plotted for the different systems, showing in general that the liquid-crystalline to gel phase transition temperature decreases under aerated conditions.

Overall, an increase in the vsCH_2_ stretch wavenumber is observed with increasing temperature for all samples (Figure 7a,b). Both planktonic and biofilm cells show cooperative events, where the wavenumber changes drastically. In contrast with the DPH fluorescence anisotropy results, the level of chain disorder does not increase significantly at 37 °C in the presence of carotenoids under aerated conditions (See Figure 7a,b at 37 °C). This indicates that, although the lipid bilayer core mobility decreases drastically with increased carotenoid content, as indicated by DPH fluorescence anisotropy (Figure 6d), the lipid acyl chains remain relatively disordered.

In the gel phase regime, acyl chain disorder is significantly higher in the presence of carotenoids (Figure 7a,b). The presence of carotenoids induced by aeration keeps the membranes highly disordered in the gel phase regime. This is likely caused by the diaponeurosporenoic chain in STX, as well as the free diaponeurosporenoic acid that disrupts lipid packing in the gel phase regime [7,31]. The destabilization of the gel phase in the presence of carotenoids leads to a strong decrease in the gel to liquid-crystalline phase temperature (Figure 7c,d) for both planktonic and biofilm growth states under aerated conditions. The results point to an unusual result. The presence of carotenoids appears to stabilize the liquid-crystalline phase even though this phase has increased rigidity. This is explained by the effects of carotenoids in the gel phase regime, where a very large increase in acyl chain disorder occurs at 15 °C. This disordering effect in the gel phase is the dominant phenomena which therefore leads to a depression of the phase transition temperature.

## 3. Discussion

### 3.1. Regulation of Lipid Composition in S. aureus Biofilms and Planktonic Cells

S. aureus biofilms play an important role in chronic infections, in particular hospital-acquired *S. aureus* infections related to the use of contaminated catheters or implants. Biofilms are resilient to different treatments and stress factors, making them difficult to treat effectively [35,36]. It is known that the lipid composition of *S. aureus* membranes can influence its ability to resist the lytic activity of antimicrobial peptides. In particular, the increased level of lipid packing induced by the presence of carotenoids in the membrane leads to increase resilience to antimicrobial peptides such as the human neutrophil defensin-1 [15]. Cardiolipin has also been shown to increase resilience to cationic antimicrobial peptide activity [21], and acidic lipopeptides such as daptomycin [17], due to its role as an inhibitor of pore formation [23]. Carotenoids are also critical in protecting the cell from oxidative stress due to the emergence of reactive oxygen species generated as an immune response [14,37]. Understanding how variations in the main lipid components of *S. aureus* membranes are modulated in response to environmental factors may be useful in identifying under what circumstances will *S. aureus* be susceptible or resistant to these defense mechanisms.

In a previous publication, catheter-induced *S. aureus* biofilm formation was shown to strongly influence lipid composition [6], strongly inhibiting the biosynthesis of carotenoids. The present work shows that the lack of aeration in the medium is responsible for inhibiting carotenoid production in *S. aureus* biofilms, and that low aeration levels also inhibit carotenoid production in planktonic cells. This regulation is in line with a recently reported AirSR oxygen detection system in *S. aureus*, which regulates oxygen-sensing and redox-signaling, and which upregulated the biosynthesis of STX in response to oxidative stress [14]. In this previous publication, the AirSR signaling system was shown to positively regulate the STX biosynthetic pathways via operon crtOPQMN. Figure 3b shows that oxygen-restricting growth conditions lead to the accumulation of the precursor 4,4′-DPE, which indicates that CrtN is downregulated at low oxygen levels. It is also worthwhile noting that, overall, there is a higher accumulation of STX biosynthesis species in planktonic cells compared to biofilms, indicating higher levels of lipid metabolism in the planktonic growth condition (Figure 3b). In addition, the non-glycosylated 4,4′-DNPA species accumulates in biofilms indicating that the completion of the biosynthesis of STX may be due to the lack of accessible glucose in biofilms.

Regarding phospholipid composition, the present results show that cardiolipin appears in high proportions in both aerated and non-aerated biofilms (Figure 4b). This molecule is known to increase mechanical strength and induce an increase in membrane rigidity [21,23]. Its increased presence in *S. aureus* biofilms is likely to increase the resistance of biofilm cells to osmotic stress [38], and acute acid stress [39], in addition to its role as inhibitor of antimicrobial peptide activity [21,40]. In contrast to carotenoids, phospholipid composition does not appear to be very sensitive to aeration levels in biofilms. Instead, planktonic cells do show a pronounced increase in Lysyl-PG under oxygen-restricting growth conditions. Lysyl-PG is a cationic lipid that changes the surface electric potential of *S. aureus* membranes. It has been shown to act as a defense mechanism of cationic antimicrobial peptides by reducing the binding affinity of the peptides to the membrane surface [41]. 

### 3.2. Variation in the Biophysical Properties of S. aureus Membranes

A variety of physical techniques were used to assess how the biophysical properties of *S. aureus* membranes change in biofilms and planktonic cells under oxygen-restricting or aerated growth conditions. The physical aspects that were explored were lipid headgroup spacing as measured with Laurdan fluorescence GP, mobility in the membrane core as measured with DPH fluorescence anisotropy, and lipid acyl chain order as measured with FTIR spectroscopy. Thermotropic measurements were performed in order to observe the behavior of the membrane in the gel phase regime at low temperatures and the liquid-crystalline regime at high temperatures, and also to assess gel to liquid-crystalline phase transition temperatures, which are important indicators of the membrane physical state [6,8,10,11]. Downward shifts in the phase transition temperature indicate that the liquid-crystalline phase of the membrane is stabilized with respect to the gel phase.

Under aerated growth conditions, headgroup spacing was observed to decrease in planktonic cells (Figure 4). indicating a more condensed membrane. However, the changes in headgroup spacing were not significant in biofilms when varying aeration levels (Figure 4c,d). In contrast, the level of lipid bilayer mobility at the core decreases significantly for both biofilms and planktonic cells in the presence of carotenoids (Figure 5). These results are consistent with previous publications [31,42]. It is interesting to compare these results with those found via FTIR. At 37 °C, the acyl chain order does not vary significantly in the presence of carotenoids. This is in sharp contrast with the DPH fluorescence anisotropy results. A possible explanation for this is that the presence of carotenoids somehow maintains acyl chain disorder but keeps low internal mobility. This could be explained by the fact that the 4,4′-DNPA present in STX spans a distance longer than the thickness of the bilayer. This may imply that the molecule may anchor the leaflets without perturbing the acyl chain order very much but reducing internal mobility. An angled orientation of polar xanthophylls to fit into the membrane geometry has been reported [43]. This angled configuration may lead to disorder in the lipid acyl chains, while increasing the structure of the lipid bilayer core.

It is important to notice that in the gel phase regime, acyl chain order is significantly affected by the level of aeration (Figure 6), showing a sharp decrease in acyl chain order at 15 °C (Figure 6 and Table 3). This coupled to a sharp decrease in the gel to liquid-crystalline phase transition temperature (Table 3), indicating that the gel phase is destabilized when carotenoids are abundant. This result is important due to the fact that the more disordered liquid-crystalline phase is considered the physiologically relevant phase due to its increased mobility. By making the gel phase more disordered, carotenoid production may help the bacteria maintain a physiologically active membrane at low temperatures [8,10]. A recent study by Seel et al. showed that carotenoids regulate membrane fluidity in Staphylococcus xylosus, in particular greatly affecting the fluidity of the membrane at low temperatures [32]. The authors argue that this may be a mechanism for thermoregulation of membrane properties, helping the bacterium to maintain highly fluid membranes at low temperature growth conditions. Table 3 presents an integration of all the biophysical observations using the different techniques described here. The variations of biophysical parameters are relevant in the susceptibility of bacteria to membrane active antimicrobial agents. For example, the activity of phospholipase A_2_ type IIA has been shown to be closely linked to lipid phase behavior and lipid packing in *S. aureus* [11], and the activity of antimicrobial peptides has been shown to be sensitive to changes in lipid packing induced by the presence of STX [6,15]. 

It is clear when comparing Figure 6c,d that biofilm membranes in general have higher phase transition temperatures than planktonic cells. Comparing the reported melting temperatures in Table 3 for the different growth conditions, an increase of 3 °C in the *T_m_* is observed in aerated biofilms compared to aerated planktonic cells, and an increase of 6 °C is observed in non-aerated biofilms compared to non-aerated planktonic cells. Pure cardiolipin has phase transition temperatures that are significantly higher than PG lipids, having equal chain lengths [44]. For example, while DMPC has a phase transition temperature at 24 °C, CL, which also 14 carbon saturated acyl chains, has a phase transition temperature around 47 °C. Since the proportion of cardiolipin increases significantly for *S. aureus* grown as biofilms (Figure 4b), this increase in CL likely explains the upward shift in T_m_. This implies that biofilm cells have more rigid lipid bilayers, compared to planktonic cells. This could be correlated with the higher rigidity of biofilm structures related to the presence of exopolysaccharides and the harsher osmotic conditions that can be found in biofilms [45].

## 4. Materials and Methods

### 4.1. Materials

The fluorescent probes LAURDAN (6-Dodecanoy-l2-dimethylaminonaphthalene) and DPH (diphenylhexatriene) were purchased from Molecular Probes (Eugene, OR, USA). HPLC-grade methanol (MeOH), chloroform (CHCl3), acetonitrile, 2-propanol, and ethyl acetate were purchased from Honeywell (Charlotte, NC, USA), while methyl tertbutyl ether (MTBE) was purchased from J.T. Baker (Palo Alto, CA, USA). LC-MS-grade ammonium acetate was purchased from Honeywell Fluka (St. Louis, MO, USA). Powder components for Luria-Bertani medium, and butylated hydroxytoluene (BHT) were purchased from Sigma-Aldrich (St. Louis, MO, USA). HPLC-grade water was obtained from a water purification system, Heal Force Smart-Mini (Shanghai, China). β-carotene (99%) with Lot number S18A010 was purchased from Alfa Aesar (Madrid, Spain).

### 4.2. Bacterial Cultures

The *S. aureus* clinical isolate strain SA401 was used in this work [8]. The isolate was recovered from the stock that is kept stored at −80 °C. In sterile conditions, an amount of the inoculum was streaked over LB agar plates. The plate was incubated at 37 °C for at least 16 h to obtain visible single colonies. After the colonies were visible, a single colony was taken and added to 10 mL of LB medium with constant agitation at 250 rpm and 37 °C for 18 h. Then, an aliquot of 10 μL was seeded in 150 mL of fresh LB in aerobic conditions or in 300 mL in oxygen-restricted conditions. The new growth was incubated for 24 h into a shaker at 250 or 150 rpm for aerobic or oxygen-restricted conditions, respectively. This study also had the objective of comparing biofilms with planktonic cells grown under different agitation conditions (A, −A). 

### 4.3. Gravity Fed System for Biofilm Growth under Aerobic and Anaerobic Conditions

For biofilm growth, three clinical catheters by Baxter (MRC0002mp) were used, two Schott Duran bottle glass of 1 L, two Duran Erlenmeyer flask of 2 L, 10 mL syringes, microfluidics connectors, a magnetic stir bar, and a magnetic stir plate. Connecting three clinical catheters with the microfluidic connectors, the Schott bottle with 1L of LB medium was placed higher than the empty bottle. Both bottles have a rubber stopper to avoid contamination; the flow was then started. Then, 10 mL of growth liquid was introduced via syringe into the injection port of the catheter and the flow was stopped for at least 2 h to promote cell adhesion; the catheter must be incubated at 37 °C. Afterwards, the flow was restarted at a rate of 1 drop/min and incubated for 48 h. The difference between aerated and non-aerated conditions were controlled through stirring of the feeding medium. For this, two Erlenmeyer flasks of 2 L each were used, and a sterile magnet was introduced to the Erlenmeyer flask with fresh medium, and it was placed on the magnetic stirrer under constant agitation (350 rpm).

### 4.4. Separation of Total Lipids

For lipid extraction, 100 mg of lyophilized cells were previously weighed, macerated, and added in glass tubes with 10 glass beads. They were dissolved in a solution of MeOH:CHCl_3_ (2:1); 10 mL of CHCl_3_ and 20 mL of MeOH, and vortex-mixed for 5 min. After this time, 10 mL of CHCl_3_ was added to make the ratio 1:1 and vortex-mixed for 1 min. Finally, 10 mL of saline solution (1.7 M NaCl) was added and then cells were vortexed every 15 min for 4 h to obtain a homogeneous solution. The sample was centrifuged at 750× *g* at 4 °C for 15 min. After centrifugation, the phase separation was achieved, and it was possible to observe three different organic phases; the thick layer of proteins was removed using a syringe and the CHCl_3_-containing lipids were gently aspirated and added into a new glass vial. The lipids were dried with nitrogen, lyophilized, and weighted.

### 4.5. Scanning Electron Microscopy (SEM) of Biofilms and Planktonic Cells

After 48 h, a section of the catheter was cut and split open by cutting laterally; for planktonic cells, 1 mL of overnight was centrifuged to obtain the pellet. The samples were fixed for 24 h with glutararaldehyde 2.5%. Therefore, the samples were first rinsed to remove the salts with HPLC-water, then they were submerged in a series of mixtures of 70%, 95%, and 100% ethanol for 30 min each, and finally, the samples were covered with gold. ImageJ software (1.53t) was used to measure cell areas; for this, the bar included in the picture of 2 µm was useful for the transformation into pixels, and in this way, it was possible to measure the area, assuming a circle for the bacteria figure. A total of 20 cells of 5 pictures for each sample (biofilm A, −A and planktonic A, −A) were measured.

### 4.6. Fluorescence Spectroscopy

To perform measurements, it was necessary to form multilamellar vesicles. From bacterial lipid extracts, 1 mg were dissolved in MeOH:CHCl_3_ (1:9, *v*/*v*) with LAURDAN or DPH at a proportion 1:150 (probe to lipid ratio). The solution was dried under nitrogen flow and lyophilized for at least 10 h to remove residual CHCl_3_. After this time, the samples were rehydrated with 1 mL of buffer, and five cycles of vortex and heat were repeated to obtain multilamellar vesicles. During the preparation, an additional step was added due to the difficulty in detaching the sample; thus, cycles of tip sonication were used to help homogenize the sample. All measurements were taken from 10 to 45 °C using 30 μM of lipid in 1 mL of buffer in a cuvette. Fluorescence spectroscopy was performed using a PC-1 ISS photon-counting spectrofluorometer that has a temperature controller and continuous magnetic stirring to maintain homogeneity in the samples. 

Laurdan has a low solubility in water; it is used as a sensitive probe to study the level of hydration of the headgroup acyl chain interphase of the membrane. Laurdan properties have been described using the generalized polarization GP that is calculated using [46] the following equation:

(1) $$GP=\frac{I_{B}-I_{R}}{I_{B}+I_{R}}$$

where $I_{B}$ (intensity at 440 nm) and $I_{R}$ (intensity at 500 nm) are the measured fluorescence intensities. When Laurdan is in the membrane, it exhibits a red shift of the emission spectrum as the membrane changes from gel to liquid-crystalline phase; the factor GP can be used to measure this shift. For the measurements, the samples were excited at 350 nm and the emission was recorded at 440 and 500 nm. On the other hand, using DPH, we can measure the anisotropy which provides information on the dynamic behavior of the lipid bilayer core. For DPH, it is necessary to use polarized light, and this will result in a selective excitation of those molecules whose absorption transition dipole is parallel to the electric vector; samples were excited at 350 nm and the fluorescence intensity was monitored at 428 nm; anisotropy is defined by [46]

(2) $$R=\frac{I_{\;∥}-I_{⊥}}{I_{\;∥}+{2I}_{⊥}}$$

where is $I_{∥}$ the vertically polarized fluorescence intensity and $I_{⊥}$ the perpendicularly polarized fluorescence intensity.

### 4.7. Fourier Transform Infrared (FTIR) Spectroscopy

The experiments were prepared on a BioATR II cell integrated to a Tensor II spectrometer (Bruker Optics, Ettlingen, Germany) with a liquid nitrogen MCT detector using a spectral resolution of 4 cm^−1^ and 120 scans per spectrum. The temperature range was set by a computer-controlled circulating water bath Huber Ministat 125 (Huber, Offenburg, Germany). All the experiments were carried out with a heating rate of 1 °C/min and a stabilization time of 120 s at each temperature. First, the background was taken using buffer (HEPES 20 mM, 500 mM NaCl, and 1 mM EDTA) from 10 to 45 °C. Subsequently, the biofilms extracts were deposited on the silicon crystal (0.3 mg) to prepare, in situ on the BioATR II cell, the solid-supported lipid bilayers, where they were hydrated with 20 µL of buffer at 37 °C for 10 min and measurements were recorded at each temperature of the ramp. Finally, to determine the position of the vibrational band in the range of the second derivative of the spectra, all the absorbance spectra were cut in the 2970–2820 cm^−1^ range, shifted to a zero baseline, and the peak picking function included in OPUS software 7.5. The results were plotted as a function of the temperature using the OriginPro 8.0 software (OriginLab Corporation, Northampton, MA, USA). To determine the melting temperature T_m_ of the lipids, the curve was fitted according to the Boltzmann model to calculate the inflection point of the obtained thermal transition curves using Python (3.11.3).

### 4.8. Targeted Lipidomics Analysis by LC-MS/MS

In this research, three families of lipids from *S. aureus* PG, Lysyl-PG, and CL were analyzed with LC-MS/MS. A Dionex UltiMate 3000 Ultra-high-performance liquid chromatographer (UHPLC) equipped with an online degasser, binary pump, autosampler, and a thermostated column compartment coupled with an LCQ Fleet Ion Trap Mass Spectrometer through an ESI source operated in positive and negative mode was employed (Thermo Scientific, San Jose, CA, USA). Raw lipids data were acquired and processed using the Xcalibur 4.3 software (Thermo Scientific, San Jose, CA, USA). The dried extracts from *S. aureus* and standards were dissolved at 10 ppm in CHCl_3_:MeOH (1:2) and analyzed using 10 μL injection volume of the samples kept at 15 °C (in the autosampler) on a C18 column at 50 °C (Phenomenex Kinetex C18, 2.1 × 50 mm, 2.6 μm particle size) protected with a SecurityGuard Cartridge Phenomenex C18 (4 × 2 mm, 3 μm particle size) pre-column. The chromatographic separation was carried out, employing as solvent A acetonitrile:ammonium formate 10 mM (6:4) and 2-propanol:acetonitrile (9:1) as solvent B. The gradient elution, at a flow of 300 µL/min, was as follows: a 45% B hold for the first 2 min, then from 45 to 65% B in 18 min, successively from 65 to 100% B in 11 min, maintaining isocratic at 100% B for 4 min, then from 100 to 45% B in 2 min and maintained at 45% B for 5 min (total running time: 40 min), an optimization of previously published conditions [47]. The parameters of the ESI source were as follows for the positive ionization mode ESI(+): sheath gas flow rate at 15 (arbitrary units); spray voltage at 4.50 kV; capillary temp at 330 °C; capillary voltage at 48 V; tube lens at 105 V. For ESI(−), conditions were as follows: sheath gas flow rate at 15 (arbitrary units); spray voltage at 5.50 kV; capillary temp at 330 °C; capillary voltage at −47 V; and tube lens at −85.6 V. Mass spectra were acquired in full ion scanning over a mass range *m*/*z* 110–2000, and tandem mass analysis, using automated data-dependent MS/MS with a 30% collision energy, was used to obtain the corresponding fragment ions with an isolation amplitude of 3 *m*/*z*.

### 4.9. Carotenoids Analysis by LC-DAD-APCI-MS/MS

The identification and quantification of carotenoids were performed using a method previously published, with some modifications [8]. Dried extracts were resuspended at 10 ppm in MeOH:CHCl_3_ (2:1) and analyzed with liquid chromatography (LC) using an UHPLC Dionex UltiMate 3000 equipped with diode-array detector (DAD) coupled to a LCQ Fleet Ion Trap Mass Spectrometer (MS) employing an atmospheric pressure chemical ionization (APCI) source operated in negative mode. Raw data were acquired and processed using Xcalibur 4.3 software. Both equipment and software were obtained from the brand Thermo Scientific, San Jose, CA, USA. The chromatographic separation was performed at room temperature with 10 μL injection volume of samples kept at 5 °C on a YMC-C30 column (150 × 4.6 mm i.d., 3 μm particle size; YMC America, Inc., Devens, MA, USA) protected with a SecurityGuard Cartridge Phenomenex C18 (4 × 2 mm, 3 μm particle size) pre-column. The mobile phases contained 400 mg/L of ammonium acetate dissolved in a mixture of methanol:methyl tert-butyl ether:water (80:18:2 *v*/*v*/*v*, for solution A and 8:89:3 *v*/*v*/*v*, for solution B). The elution gradient at a constant flow rate of 450 μL/min was as follows: 5% B for the first 3 min, then from 5 to 10% B in 6 min, from 10 to 25% B in 10 min, from 25 to 40% B in 4 min, maintaining isocratic elution at 40% B for 4 min, then from 40 to 100% B in 4 min and constant 100% B for 3 min. Reconditioning from 100 to 5% B in 2 min and maintaining isocratic conditions for additional 4 min, with a total running time of 40 min per sample. The DAD was performed over the entire UV–vis range (240–600 nm), and the characteristic absorbances of the carotenoids were extracted at 450 nm and STX precursor at 286 nm. The APCI source was operated with the following parameters: vaporizer temperature, 300 °C; discharge current, 15.0 μA; capillary voltage, −25.0 V; tube lens, −80.0 V; capillary temperature, −350 °C; envelope gas flow, 50 arbitrary units; auxiliary gas flow, 30 arbitrary units. In addition, the ion trap was configured to operate in full scan (*m*/*z* 65–1200) and data-dependent MS/MS at 30% collision energy to obtain the corresponding fragment ions with an isolation amplitude of 3 *m*/*z*.

For the quantification of *S. aureus* carotenoids, there were no commercially available standards, so β-carotene was used as an external standard (Figure S2). Quantitative analysis was realized with a calibration curve over the range of 5–500 μg/mL, using the operating conditions described above. Limits of detection (LODs) and quantification (LOQs) corresponded to 1.5 and 5.0 µg/mL, respectively. Additionally, a molecular weight correction factor was applied to account for the difference in detector response, consistent with previous reports [48,49]. The linear regression curve of the standard was y = 2512.4x – 4352.6; *R*^2^ = 0.999. Carotenoids concentration is displayed in μg/g dry cell weight. 

### 4.10. Statistical Analysis

The different experiments were carried out with three biological replicates. Error bars represent the standard error of the mean (mean ± SD). Data obtained were subjected to analysis of variance (ANOVA) and the means were compared with Tukey’s HSD using software Prism—GraphPad 8.0.2. Results with *p*-value (*p* ≤ 0.05) were considered statistically significant.

## 5. Conclusions

*S. aureus* is a major bacterial human pathogen, and a leading cause of a variety of clinical conditions including bacteremia and infective endocarditis, as well as osteoarticular and skin and soft tissue infections. The emergence of methicillin-resistant strains has led to the search for alternative treatment strategies that include antimicrobial peptides and oxidative agents. However, these treatment strategies are sensitive to the composition and physical properties of *S. aureus* membranes, which can vary due to growth conditions. Here, we show that oxygen-restricting growth conditions decreases carotenoid content in *S. aureus* biofilms and planktonic cells. Changes in phospholipid composition are also observed, where cardiolipin is found to increase in proportion to other phospholipids in biofilms, and Lysyl-PG is found to increase under oxygen-restricting growth conditions in planktonic cells. These changes in composition lead to variations in the biophysical properties of the membranes. Under oxygen-restricting conditions, the reduction in carotenoids leads to a decrease in order at the membrane core. However, this decrease in membrane order is not tied to an increase in acyl chain order of the phospholipids, indicating that the presence of carotenoids may rigidify the membrane while maintaining disorder chains in the phospholipids. At low temperatures, oxygen-restricting conditions lead to a sharp increase in membrane order. This indicates that the presence of carotenoids maintains high levels of fluidity in *S. aureus* membranes at low temperatures. These results are relevant in understanding the physical state of the membrane under different environmental conditions, which vary in oxygen levels. This can lead to a better understanding of the susceptibility of *S. aureus* to different membrane active antimicrobial agents under different environments. In particular, certain tissues are characterized by having low oxygen levels, which can lead to reduced levels of carotenoids in *S. aureus* cells residing in these tissues.

## Figures and Tables

**Table 1:** Mean area of *S. aureus* cells measured using scanning electron microcopy images for the different growth conditions. Non-aerated cells are observed to be smaller than aerated cells for biofilms and planktonic growth conditions.

Growth Condition	$\mathrm{Average}\;\mathrm{Cell}\;\mathrm{Area}\;[µm^{2}\pm S.D.]$
Aerated biofilms	0.41 ± 0.12
Non-aerated biofilms	0.29 ± 0.05
Aerated planktonic cells	0.37 ± 0.04
Non-aerated planktonic cells	0.28 ± 0.04

**Table 2:** Concentrations of carotenoids and carotenoids precursor in biofilms and planktonic *S. aureus* cells.

No.	Compound	Biof-Aero	Biof-NoAero	Plant-Aer	Plant-NoAer
		(μg/g Dry Cell Weight ± SD)			
1	4,4-DPE-Iso1	556.72 ± 22.29	1129.74 ± 56.27	67.36 ± 2.51	4069.07 ± 125.34
2	4,4-DPE-Iso2	42.78 ± 3.45	252.12 ± 7.31	n.d.	346.36 ± 3.85
3	4,4-DNPA	1742.18 ± 88.96	437.63 ± 19.86	802.07 ± 20.87	111.24 ± 11.81
4	STX-Iso-1	n.d.	n.d.	n.d.	146.78 ± 6.91
5	STX-Iso-2	132.17 ± 11.52	22.06 ± 2.29	591.55 ± 32.91	n.d.
6	STX-Iso-3	64.23 ± 4.48	17.76 ± 2.16	49.72 ± 4.87	n.d.
7	STX-Iso-4	42.20 ± 2.83	n.d.	121.79 ± 4.80	n.d.
8	STX-Iso-5	n.d.	n.d.	243.92 ± 6.30	n.d.
9	STX-Iso-6	520.01 ± 36.17	119.57 ± 9.81 ^a^	2232.06 ± 112.15	41.83 ± 3.73 ^a^
10	STX-Iso-7	491.00 ± 15.36	109.58 ± 4.50	1316.68 ± 34.64	n.d.
11	STX-Iso-8	93.78 ± 4.66	n.d.	307.91 ± 5.40	n.d.
12	STX-C17	80.20 ± 5.40 ^b^	n.d.	92.29 ± 4.72 ^b^	n.d.
	Total carotenoids precursor	599.50 ± 36.34	1376.86 ± 61.70	67.36 ± 2.51	4415.43 ± 263.23
	Total carotenoids	3168.78 ± 57.73	706.59 ± 15.69	5758.00 ± 72.50	299.85 ± 53.38

Equal letters in the same rows are not significantly different, according to the ANOVA test (Tukey, *p* < 0.05). n.d. = not detected.

**Table 3:** Biophysical parameter measurements for planktonic and biofilm *S. aureus* total lipid extracts under aerated and non-aerated conditions.

Biophysical	Planktonic		Biofilm	
Membrane Parameters	Aerated	Non-Aerated	Aerated	Non-Aerated
10 °C	0.539 ± 0.001	0.498 ± 0.002	0.535 ± 0.001	0.545 ± 0.001
Laurdan GP				
37 °C	0.327 ± 0.006	0.168 ± 0.001	0.274 ± 0.004	0.233 ± 0.002
10 °C	0.231 ± 0.006	0.189 ± 0.008	0.259 ± 0.004	0.234 ± 0.014
DPH Anisotropy				
37 °C	0.178 ± 0.008	0.096 ± 0.006	0.181 ± 0.003	0.143 ± 0.007
10 °C	2851.36	2850.74	2852.08	2851.20
${CH}_{2}$ Assy. Strech				
37 °C	2853.11	2853.21	2852.97	2853.04
$T_{m\;}$ [°C]	12	18	15	24

## Data Availability

The data involved in this paper have been presented in articles and supporting materials in the form of diagrams or tables.

## Supplementary Materials

The following supporting information can be downloaded at: https://www.mdpi.com/article/10.3390/ijms241914906/s1.
