# Supplementary material for: Staphylococcus aureus Modulates Carotenoid and Phospholipid Content in Response to Oxygen-Restricted Growth Conditions, Triggering Changes in Membrane Biophysical Properties
Source: Int J Mol Sci. 2023 Oct 5;24(19):14906. doi: 10.3390/ijms241914906 (PMC10573160; doi:10.3390/ijms241914906)
Supplement: Supplementary file 1 [file ijms-24-14906-s001.zip › ijms-2575795-supplementary.pdf]

# *Staphylococcus aureus* Modulates Carotenoid and Phospholipid Content in Response to Oxygen-Restricted Growth Conditions, Triggering Changes in Membrane Biophysical Properties

**Laura Zamudio-Chávez [1], Elizabeth Suesca [1], Gerson-Dirceu López [2], Chiara Carazzone [3], Marcela Manrique-Moreno [4] and Chad Leidy [1,*]**

[1] Biophysics Group, Physics Department, Universidad de los Andes, Bogotá 111211, Colombia; ld.zamudio@uniandes.edu.co (L.Z.-C.); e.suesca87@uniandes.edu.co (E.S.)
[2] PhysCheMath Research Group, Chemistry Department, Universidad de América, Bogotá 111211, Colombia; gerson.lopez@profesores.uamerica.edu.co
[3] Laboratory of Advanced Analytical Techniques in Natural Products (LATNAP), Chemistry Department, Universidad de los Andes, Bogotá 111211, Colombia; c.carazzone@uniandes.edu.co
[4] Chemistry Institute, Faculty of Exact and Natural Sciences, University of Antioquia, Medellin 050010, Colombia; marcela.manrique@udea.edu.co
* Correspondence: cleidy@uniandes.edu.co; Tel.: +57-1-3394949 (ext. 2741)

**Table S1**. The identified phosphatidyl-glycerol (PG), phospholipid lysyl-phosphatidylglycerol (Lys-PG), and cardiolipin (CL) in extract from *S. aureus.*

| **Phosphatidylglycerols [M-H]⁻** | **Retention Time (min)** | ***m/z*** | **Main fragments (*m/z*)** |
|---|---|---|---|
| PG (30:0) | 4.02 | 693.5 | 619, 469, 451, 377, 241, 227. |
| PG (31:0) | 4.95 | 707.5 | 483, 465, 405, 391, 255, 241, 277. |
| PG (32:0) | 5.65 | 721.5 | 648, 497, 479, 405, 269, 241. |
| PG (33:0) | 6.95 | 735.5 | 661, 511, 493, 451, 419, 283, 241. |
| PG (34:0) | 7.61 | 749.5 | 675, 525, 507, 433, 297, 269, 241. |
| PG (35:0) | 9.08 | 763.5 | 688, 539, 521, 447, 377, 311, 241. |

| **Lysyl phosphatidylglycerols [M+H]⁺** | **Retention Time (min)** | ***m/z*** | **Main fragments (*m/z*)** |
|---|---|---|---|
| Lys-PG (31:0) | 7.45 | 837.3 | 819, 802, 537, 410, 301, 283, 266. |
| Lys-PG (32:0) | 8.21 | 851.3 | 833, 816, 581, 551, 301, 283, 266. |
| Lys-PG (33:0) | 10.32 | 865.5 | 847, 830, 591, 565, 301, 283, 266. |
| Lys-PG (34:0) | 11.27 | 879.3 | 861, 844, 579, 511, 301, 283, 266. |
| Lys-PG (35:0) | 12.73 | 893.4 | 875, 856, 593, 581, 301, 283, 266. |

| **Cardiolipins [M-H]⁻** | **Retention Time (min)** | ***m/z*** | **Main fragments (*m/z*)** |
|---|---|---|---|
| CL (60:0) | 21.04 | 1296.0 | 755, 675, 647, 619, 377. |

| | | | |
|---|---|---|---|
| CL (61:0) | 21.75 | 1310.0 | 1068, 769, 647, 633, 619, 391, 377. |
| CL (62:0) | 21.99 | 1323.9 | 1081, 783, 755, 647, 619, 405, 377. |
| CL (63:0) | 22.41 | 1337.9 | 1095, 797, 89, 633, 619, 423, 405. |
| CL (64:0) | 22.74 | 1352.0 | 1109, 783, 703, 674, 647, 629, 405. |
| CL (65:0) | 23.17 | 1366.0 | 1123, 797, 688, 661, 647, 419, 405. |
| CL (66:0) | 23.36 | 1380.0 | 1137, 811, 783, 675, 647, 433, 405. |
| CL (67:0) | 23.87 | 1394.0 | 825, 784, 689, 675, 647, 447, 433. |
| CL (68:0) | 24.03 | 1408.0 | 1165, 839, 811, 703, 675, 647, 433. |
| CL (69:0) | 24.32 | 1422.0 | 1179, 825, 731, 689, 675, 447, 433. |
| CL (70:0) | 24.62 | 1436.0 | 1193, 833, 703, 689, 461, 447, 419. |

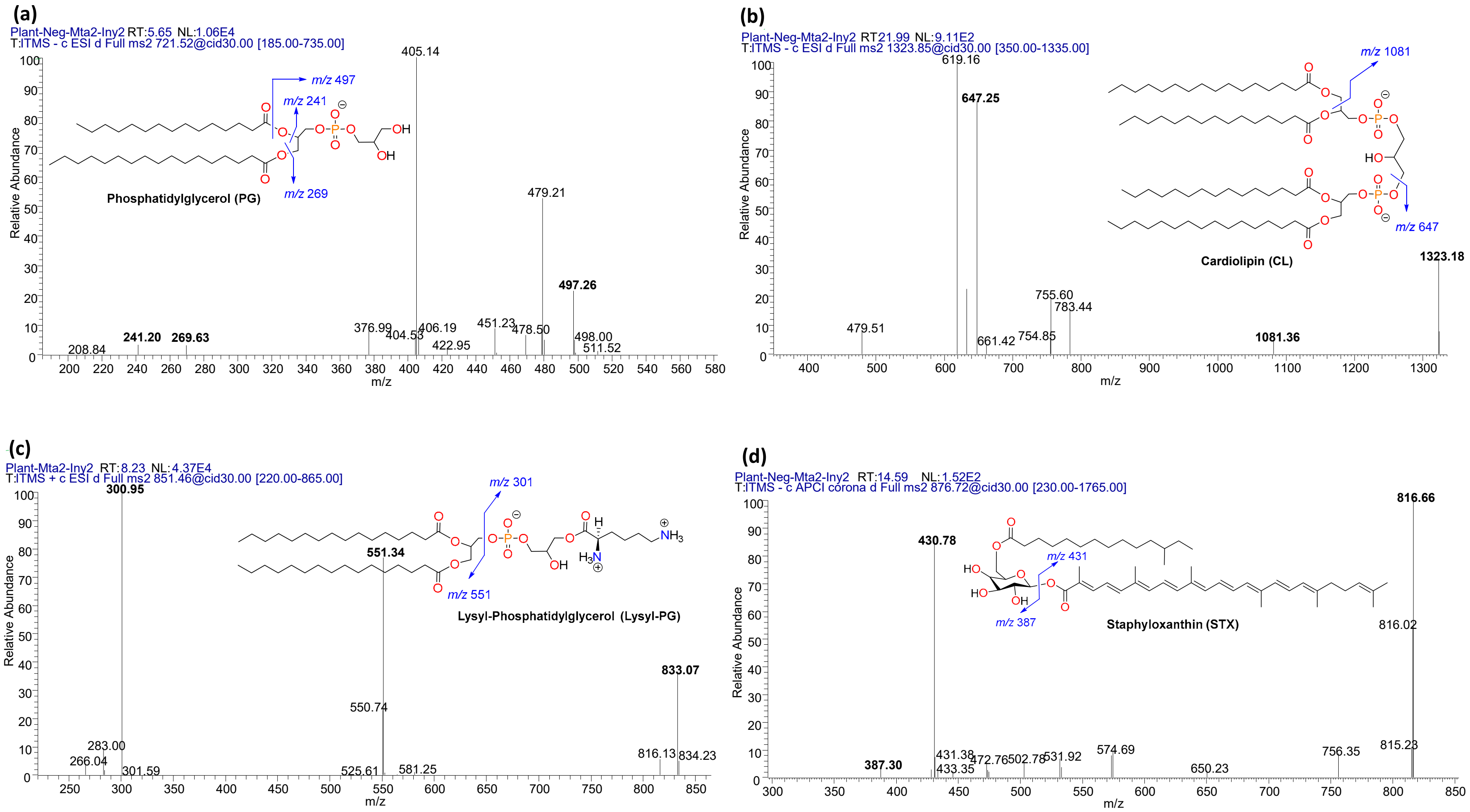


**Figure S1**. MS/MS spectra of Phosphatidylglycerol (a), Cardiolipin (b), Lysyl-Phosphatidylglycerol (c), and Staphyloxanthin (d).

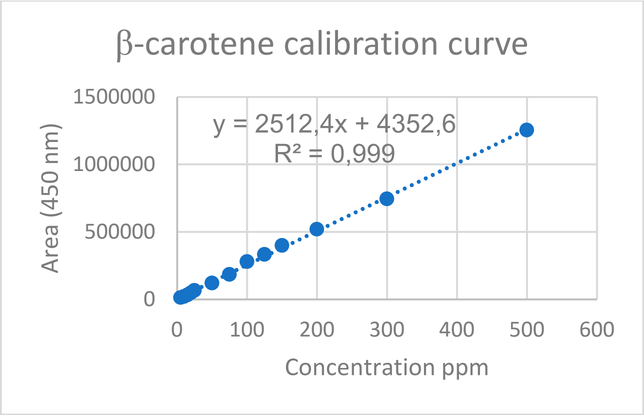


**Figure S2.** Calibration curve of β-carotene used for the quantification of S. aureus carotenoids.
